# Prothymosin-α Variants Elicit Anti-HIV-1 Response via TLR4 Dependent and Independent Pathways

**DOI:** 10.1371/journal.pone.0156486

**Published:** 2016-06-16

**Authors:** G. Luca Gusella, Avelino Teixeira, Judith Aberg, Vladimir N. Uversky, Arevik Mosoian

**Affiliations:** 1 Department of Medicine, Icahn School of Medicine at Mount Sinai, New York, New York, United States of America; 2 Department of Molecular Medicine and USF Health Byrd Alzheimer's Research Institute, Morsani College of Medicine, University of South Florida, Tampa, FL, United States of America; 3 Biology Department, Faculty of Science, King Abdulaziz University, P.O. Box 80203, Jeddah 21589, Kingdom of Saudi Arabia; 4 Laboratory of Structural Dynamics, Stability and Folding of Proteins, Institute of Cytology, Russian Academy of Sciences, St. Petersburg, Russian Federation; Vita-Salute San Raffaele University School of Medicine, ITALY

## Abstract

**Background:**

Prothymosin α (ProTα) (isoform 2: iso2) is a widely distributed, small acidic protein with intracellular and extracellular-associated functions. Recently, we identified two new ProTα variants with potent anti-HIV activity from CD8^+^ T cells and cervicovaginal lavage. The first is a splice variant of the ProTα gene known as isoB and the second is the product of ProTα pseudogene 7 (p7). Similarly to iso2, the anti-HIV activity of both variants is mediated by type I IFN. Here we tested whether the immunomodulatory activity of isoB and p7 are also TLR4 dependent and determined their kinetic of release in response to HIV-1 infection.

**Methods:**

Type I, type III, TNF-α and IL-6 mRNA inducing activity was determined in macrophages from wild type and TLR4 knockout mice treated with recombinant ProTα variants. Supernatants from mock and HIV infected cells were analyzed by mass spectrometry in positive and negative modes for the presence of ProTα variants. *In silico* structural and functional analysis of ProTα variants were performed.

**Results:**

We show that both isoB and p7 upregulate IFN-β, IFN-λ1, IL-6, TNF-α and RANTES mRNAs in primary human macrophages. The potent stimulation of IFN-β by the recombinant ProTα variants in human macrophages is dependent on the TLR4 pathway, whereas the induction of TNF-α and IL-6 may also occur independently of TLR4, suggesting the interaction of ProTα variants with other signaling molecules/receptors. In silico analyses confirmed that the novel isoB and p7 variants are intrinsically disordered proteins, which lack the NLS and mass spectrometry showed release of ProTα variants within minutes post HIV-1 infection. These features are consistent with the function of ProTα variants as damage associate molecular patterns (DAMPs).

**Conclusions:**

Our findings indicate that ProTα variants strongly inhibit viral replication mainly, but not exclusively, through TLR4 signaling and that they are released within minutes of viral infection suggesting that they may function as DAMPs.

## Introduction

In humans, one ProTα gene, located on chromosome 2, and eight ProTα pseudogenes, scattered on different chromosomes, have been described [1, 2]. ProTα produces 2 unspliced and 16 alternatively spliced transcripts (AceView; NCBI) of which only three transcripts are known to code for ProTα-isoforms 1 and 2, which differ only by an extra glutamic acid (111 amino acids (aa) and 110 aa, respectively) [2–4]. We recently isolated the isoform predicted by the splice variant CRA_b or isoB and the product of ProTα pseudogene 7 (p7 ProTα) from CD8^+^ T cells line and cervicovaginal lavages [5–7].

The new variants isoB and p7 are less abundant than of ProTα isoform 2 (iso2), which belongs to a family of small acidic, intrinsically disordered proteins with both intra- and extra-cellular localization that might be functionally linked to cell proliferation and apoptosis [6, 8]. Iso2 is not only found in supernatants of cultured cells, but also in blood and cervicovaginal lavages where it may play a role in cell-mediated immunity as well as in anti-ischemic, anti-cancer, anti-bacterial, and anti-viral functions [9–15]. IsoB and p7 are smaller than iso2, lack a NLS, and have an isoelectric point (pI) of 3.71 and 10.96, respectively. Despite the lack of signal peptide for protein secretion, all ProTα variants are found extracellularly. Each variant may possess unique activities and different regulation as indicated by the down regulation of isoB, but not p7 or iso2, during the differentiation of monocytes to dendritic cells [7].

We previously showed that iso2 ProTα contributes to the anti-HIV-1 activity of CD8^+^ T cells via the TLR4-dependent induction of type I IFN [13]. The direct interaction of iso2 with TLR4/MD2 has been recently confirmed by Ueda’s group [16]. Others also showed competition between iso2 C-terminal acidic peptide and LPS for binding to TLR4 in neutrophils [17]. While the newly isolated ProTα variants isoB and p7 share a 50% sequence homology with iso2 and possess potent anti-HIV-1 activity [7], it is unknown whether their immunomodulatory and antiviral activities are similarly dependent on TLR4 activation. Here, we show that all variants stimulate cytokine expression through TLR4. However, while the induction of IFN-β in human macrophages is strictly dependent on TLR4, the stimulation of TNF-α and IL-6 occurs also independently of TLR4, thus suggesting the interaction of the variants with signaling molecules other than TLR4.

We also show that the isoB and p7 ProTα variants are released within minutes post HIV-1 infection. The strong induction of innate inhibitors of viral replication that stimulate TLR4 suggests that the ProTα variants could be classified as DAMPs, thus supporting the notion that DAMPs may play a role in antiviral immunity.

## Materials and Methods

### Cloning of ProTα Variants and Preparation of Recombinant Proteins

cDNAs of different variants of ProTα and Tα1 were directly cloned between the BamHI and NdeI restriction sites of the pRSET A bacterial expression vector (Invitrogen, Carlsbad, CA) so that the tag and enterokinase cleavage sites were removed from the vector [7]. The correct nucleotide sequences of Tα1, iso2, isoB and p7 cDNAs cloned into pRSET A plasmid were confirmed by DNA sequencing.

Recombinant proteins were purified as reported earlier [18] and bacterial LPS was removed by triton X-114 (Sigma, St. Louis, MO) at 1% (v/v) as described by Liu et al. [19]. Endotoxin levels, measured using the Pierce LAL Chromogenic Endotoxin Quantitation Kit (detection limit 0.1 EU/ml), were <0.1 EU/μg. Synthetic p7 ProTα protein was synthesized by LifeTein, LLC (Hillsborough, NJ) and reconstituted in PBS.

### Mass Spectrometry Analysis

Recombinant proteins were resolved on PAGE and silver stained. Visible bands were cut out of the gel, sliced into small pieces, and incubated at 56°C in freshly prepared 10mM Dithiothreitol (DTT) for 1 hour. After a brief centrifugation and removal of the DTT solution, the pellet was washed in 55mM iodoacetamide in 100mM of NH_4_HCO_3_ for acetylation. The gel pieces were dried by vacuum and then soaked in a trypsin solution overnight at 37°C. Tryptic peptides were analyzed by mass spectrometry as described previously [7].

### Isolation of Human Monocyte-Derived Macrophages

Monocytes were isolated from peripheral blood mononuclear cells (PBMCs) of healthy donors from commercially purchased buffy coats from de-identified donors (New York Blood Center). Purification was performed using magnetic beads conjugated to human anti-CD14 monoclonal antibodies (Miltenyi Biotech, Auburn, CA). Purity of CD14 positive cells, as determined by FACS, was >95%. Monocytes were differentiated into macrophages following culture for 10–15 days in Dulbecco’s modified Eagle medium containing 10% FBS. Upon differentiation, cells were plated in 12-well plates and treated for 2 h with 20 ng/ml or 200 ng/ml of recombinant proteins. Recombinant Tα1 protein purified in parallel using the same protocol was utilized as a negative control. Ultra-pure LPS (InvivoGen, San Diego, CA) at concentration10 ng/ml was used as a positive control.

### RNA Extraction and RT-qPCR

Total RNA extraction and cDNA synthesis were carried out as we previously described [7, 13]. In all the RT-qPCR experiments, the numbers indicate the fold expression change of treated over control mRNA relatively to the expression levels of the reference ribosomal protein (RPS11) gene [13].

### Isolation of Mouse Macrophages

No experiments were conducted on live mice. Tissue collection (femurs) from the wild type and TLR4KO (6–10 week old mice) were approved by the Committee on the Ethics of Animal Experiments of the Icahn School of Medicine at Mount Sinai and carried out in accordance with the ‘Guide for the Care and Use of Laboratory Animals’ (NIH publication 86–23, revised 1985). Bone marrow derived murine macrophages from wild type and TLR4^-/-^ mice (provided by JM Blander, IACUC Protocol# LA11-00231) were isolated and grown in medium supplemented with 30% Macrophage colony-stimulating factor (M-CSF) as described in previous work [20]. Macrophages were treated with 200 ng/ml of different variants of ProTα or Tα1 (as a negative control), ultra pure LPS (InvivoGen) at concentration of 10 ng/ml and 50 μg/ml of PolyI:C (as a positive control for TLR3) for 2 h. RNA was extracted and RT-qPCR was performed as described in our previous work [13].

### Inhibition of TLR4 signaling pathway in primary human macrophages

Primary human monocyte-derived macrophages were plated in 96 well plates at concentration of 3x10^4^ cell/well. Next day cells were pretreated with 1 μM of the TLR4 inhibitor TAK-242 (Invivogen, San Diego, CA) for 2 h followed by treatment with 20 ng/ml ProTα variants or controls: ultra-pure LPS (10 ng/ml) or TLR3 ligand PolyI:C (50 μg/ml) (Invivogen, San Diego, CA). Cells were then either infected with VSV envelope-pseudotyped HIV-1 (MOI: 0.01) expressing the luciferase reporter gene or left uninfected. Supernatants collected at 6 h and 48 h post-infection were analyzed by ELISA for the presence of IFN-β (VeriKine-HS^TM^ kit from PBL,Piscataway, NJ; sensitivity of the assay is 1.2 pg/ml), and TNF-α and IL-6 (Mabtech, Cincinnati, OH; assay sensitivity for both IL-6 and TNF-α is 13 pg/ml). The anti-HIV-1 activity was assessed by measuring in parallel the firefly luciferase expression in the cell lysates. Luciferase values were normalized relative to the corresponding sample’s protein concentration.

### *In silico* structural and functional analysis of ProTα variants

#### Evaluation of intrinsic disorder propensity

Disorder in different ProTα variants was evaluated by a family of PONDR predictors: PONDR^®^ VSL2B, which is one of the most accurate stand-alone disorder predictors [21]; PONDR^®^ VL3, which possesses high accuracy in finding long intrinsically disordered protein regions (IDPRs) [22]; PONDR^®^ VLXT, which, although not being the most accurate predictor, provides high sensitivity to local sequence peculiarities that are often associated with disorder-based interaction sites [23]; and PONDR-FIT, which represents a metapredictor that, being moderately more accurate than each of the individual component predictors, is one of the most accurate disorder predictors [24]. In these analyses, scores above 0.5 correspond to disordered residues/regions.

#### Finding disorder-based binding sites

Potential binding sites in disordered regions can be identified by the ANCHOR algorithm [25]. This approach relies on the pairwise energy estimation approach developed for the general disorder prediction method IUPred [26]. It is based on the hypothesis that long regions of disorder contain localized potential binding sites that cannot form enough favorable intrachain interactions to fold on their own, but are likely to gain stabilizing energy by interacting with a globular protein partner [25]. The ANCHOR algorithm suggests that certain regions of a protein have significant potential to be a binding site for an appropriate but typically unidentified partner protein called ANCHOR-indicated binding sites (AIBSs).

#### Finding potential sites of post-translational modifications (PTMs)

PTM sites in different ProTα variants were identified using a unified sequence-based predictor of 23 types of PTM sites that is available at www.modpred.org [27]. Only sites predicted to be modified with high confidence (possessing score ≥0.9) were considered in this study.

#### Evaluation of interactability of human ProTα

Interactability of human ProTα/iso2 (UniProt ID: P06454) was evaluated by STRING database, an online database resource entitled Search Tool for the Retrieval of Interacting Genes providing both experimental and predicted interaction information [28]. STRING produces a network of predicted associations for a particular group of proteins. The network nodes are proteins and the edges represent the predicted functional associations. An edge may be drawn with up to 7 differently colored lines representing the existence of the seven types of evidence used in predicting the associations. A red line indicates the presence of fusion evidence; a green line–neighborhood evidence; a blue line–co-occurrence evidence; a purple line–experimental evidence; a yellow line–text mining evidence; a light blue line–database evidence; and a black line–co-expression evidence [28].

### Release of ProTα variants in response to HIV infection and PolyI:C treatment

Primary CD4^+^ T cells were isolated from peripheral blood mononuclear cells (PBMCs) of healthy donors from commercially purchased buffy coats (New York Blood Center) from de-identified donors, by positive selection using magnetic beads conjugated to human anti-CD4 monoclonal antibody (Miltenyi Biotech, Auburn, CA). Purity of CD4 positive cells, as determined by FACS, was >95%. CD4^+^ T cells were cultured in the presence of 50 U/ml IL-2 and infected with HIV-1_BaL_ at MOI of 0.01. The release of ProTα proteins was determined by ELISA in supernatants collected 24 h post-infection as described previously [7]. Human ectocervical epithelial cells immortalized by expression of human papillomavirus 16/E6E7 [29] were infected with HIV-1_BaL_ or treated with the synthetic TLR3 ligand PolyI:C and 24 h post-treatment supernatants were collected and used as control in the ProTα ELISA measurements described above.

Time course experiments were performed on PBMCs of healthy donors from commercially purchased buffy coats from de-identified donors (New York Blood Center), (10x10^6^ cells/ml) infected with HIV-1_BaL_ at MOI of 0.01 to determine the kinetic of release of the ProTα variants. The presence of ProTα variants was determined in the supernatants collected at 10, 20, 30 and 60 min post-infection by mass spectrometry analysis in positive and negative modes.

### Statistical analysis

Data from repeated experiments were averaged and expressed as means ± standard deviation. Statistical analysis was performed by using the Student’s *t* test. *P*<0.05 values were considered statistically significant.

## Results

### Recombinant ProTα variants stimulate IFN-β and IFN-λ1 in primary human macrophages

**Fig 1:**
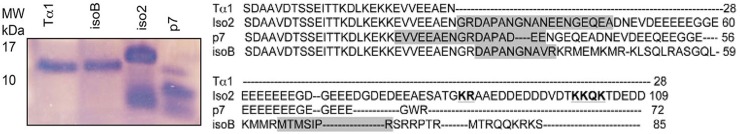
Silver stained PAGE of recombinant ProTα variants and amino acid sequence alignment of ProTα variants. Left panel: silver staining protein visualization of PAGE in which 0.5 μg of each recombinant ProTα variant was loaded. Right panel: alignment of amino acid sequence of ProTα variants. The peptide sequences identified by mass spectrometry from silver stained gel bands are in grey.

We previously showed that both native and tagged recombinant ProTα variants stimulated type I and type III interferons [7]. However, chemically synthesized p7 ProTα variant failed to show any biological activity (data not shown), suggesting that some structural hindrance may be introduced during the synthesis process. To exclude any possible interference from the tag and the enterokinase cleavage site on the activity of recombinant proteins, new recombinant proteins depleted of both signals and with sequences identical to that of the corresponding native forms were produced in bacteria (left panel of **Fig 1**) and their functional properties were tested on primary human macrophages. Electrophoresis of the recombinant proteins on polyacrylamide gel produced bands of the expected size, as well as extra bands in the case of iso2 and p7. The presence of extra bands has been previously described for iso2 ProTα as an effect of aggregation due to the highly acid nature of the iso2 protein [30, 31].

To rule out the presence of any contaminant from the recombinant protein preparations, the extra bands in the iso2 and p7 lanes were excised from the silver stained gel and analyzed by mass spectrometry. Mass spectrometry analyses exclusively revealed the presence of peptides corresponding to iso2 and p7 recombinant proteins (Right panel of **Fig 1**, grayed sequence), thus confirming the purity of the recombinant protein preparations.

**Fig 2:**
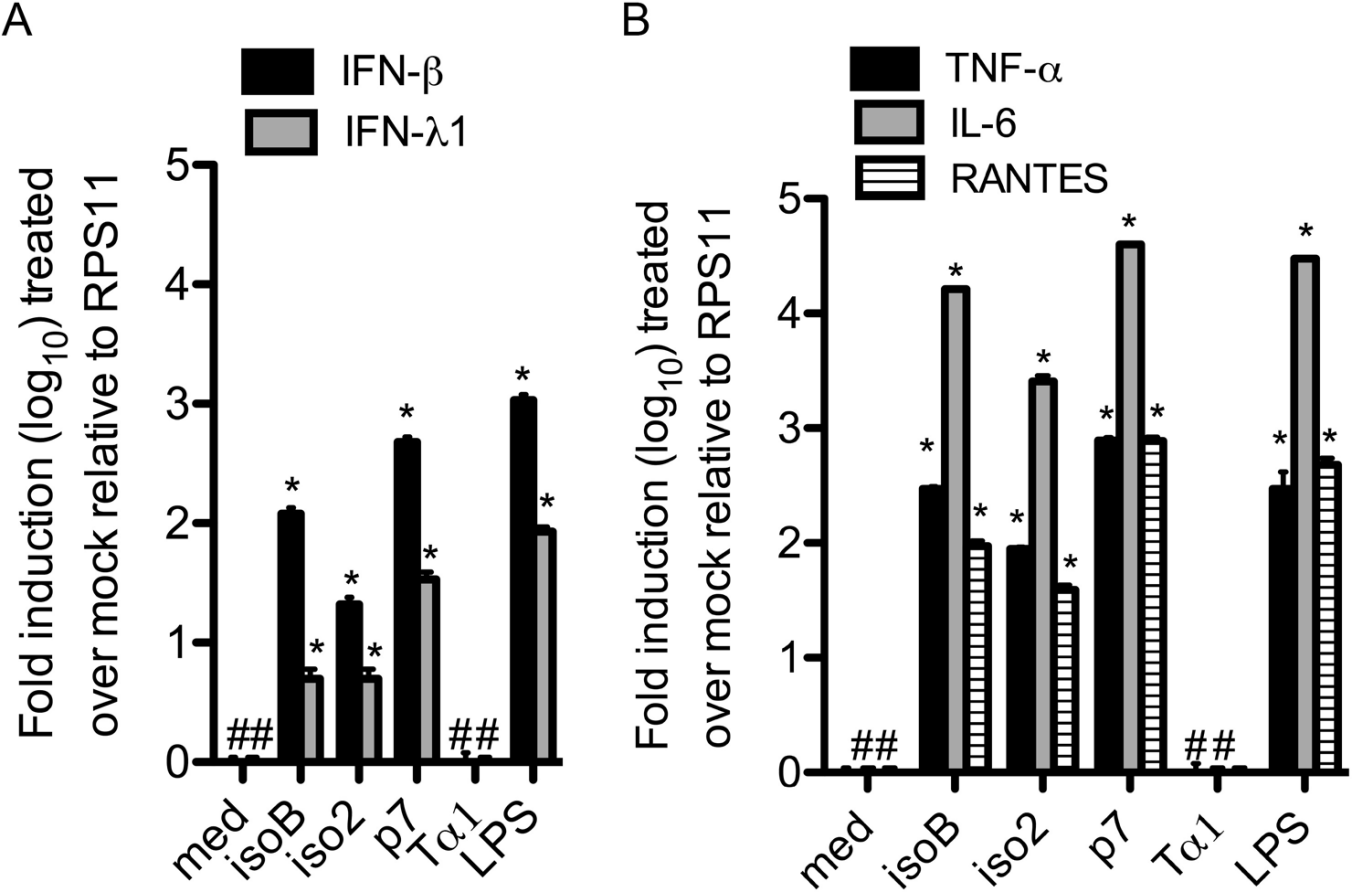
Up-regulation of mRNA of different innate inhibitors of viral replication in primary human macrophages treated with ProTα variants. A) INF-β and IFN-λ1 and (B) TNF-α, IL-6 and RANTES mRNAs induction by ProTα variants. RT-qPCR with specific primers for indicated genes was performed as detailed in Methods on cDNA from primary human macrophages treated for 2 h with 200 ng/ml of ProTα variants or LPS. mRNA transcripts levels were normalized relatively to the expression of the ribosomal protein (RPS11) control mRNA. Each experiment was done at least three times. Data represent mean ± SD. Student’s *t* test was used to compare means of treated versus control samples. *P < 0.05 was considered to be statistically significant.

In all our RT-qPCR experiments we used the different recombinant proteins at a final concentration of 200 ng/ml, as this showed a potent inhibition of HIV-1 replication in primary human macrophages in our previous studies [7]. Treatment of human primary macrophages with each recombinant ProTα variant for 2 h potently induced type I and type III interferon mRNAs. While all variants were effective, they displayed different potency, with p7>isoB>iso2 in the induction of both IFN-β (478±50, 120±15, and 21±3 fold, respectively) and IFN-λ1 (34±5 fold, 5±1, and 5±1, respectively) (**Fig 2A**). To further characterize the immunomodulatory response to individual variants, we also tested the stimulation of TNF-α, RANTES and IL-6 mRNAs. In agreement with our published data, all three recombinant ProTα variants induced the expression of TNF-α and IL-6 as well as of RANTES in human primary macrophages with the same order of potency, p7>isoB>iso2 (TNFα: 784±50, 296±3, and 89±3 fold, respectively; IL-6: 40x10^3^±35, 16x10^3^±170, and 2.5x10^3^±300 fold, respectively; RANTES: 772±70, 94±10, 39±4 fold respectively) (**Fig 2B**) [13]. Importantly, the recombinant Tα1 protein, which was purified using the same methodology in parallel with the other variants, did not show any activity, further ruling out the effect of contaminating endotoxin in the preparation of the recombinant proteins.

Overall, these results indicated that the bacteria-produced recombinant ProTα variants maintained a biological activity and suggest that the loss of activity in the synthetic proteins is caused during the chemical synthesis process [7].

### Stimulation of IFN-β by ProTα variants is TLR4 dependent, while activation of TNF-α and IL-6 might involve also other receptor(s)

**Fig 3:**
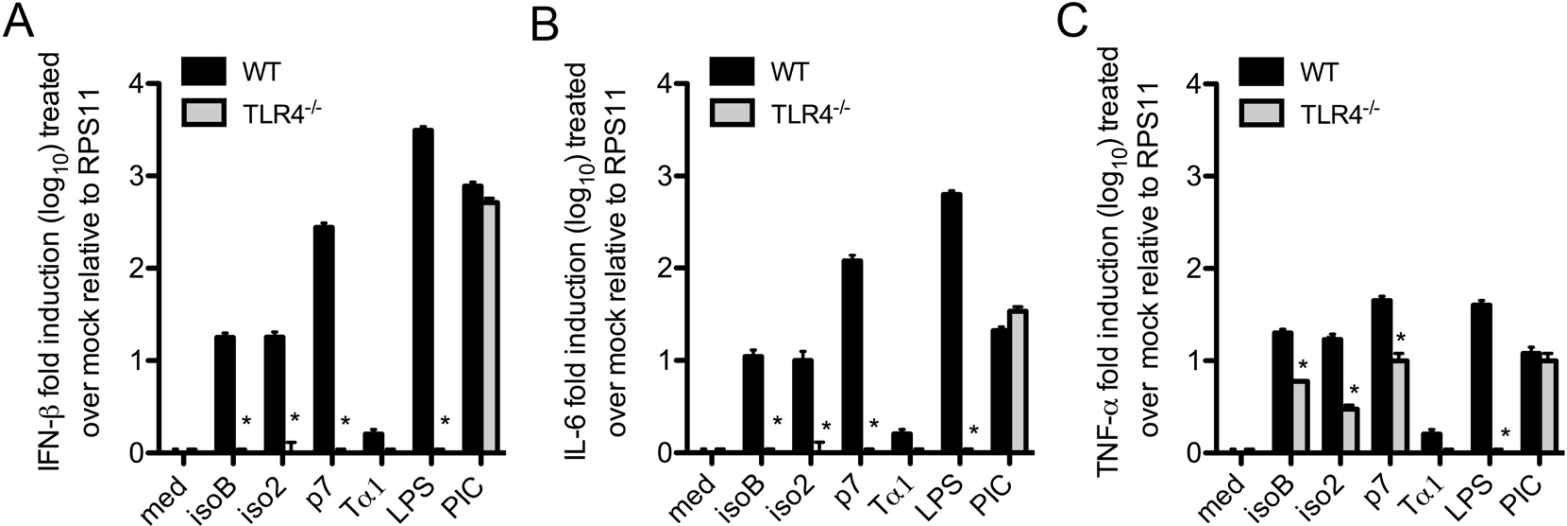
TLR4-dependent stimulation of IFN-β by ProTα variants. (A) IFN-β, (B) IL-6, and (C) TNF-α expression was measured by RT-qPCR on cDNAs from wild type and TLR4^-/-^ mouse bone marrow-derived macrophages treated with 200 ng/ml of different ProTα variants for 2 h. Though at lower levels, TNF-α remains inducible by the variants in TLR^-/-^ murine macrophages, suggesting the possible involvement of other receptor(s).

We previously demonstrated that the anti-HIV-1 activity of iso2 was mediated by type I IFN through the activation of TLR4 [13]. The isoB and p7 ProTα variants share only 50% homology with iso2 ProTα. To determine whether type I interferon and TNF-α inducing activities of ProTα variants are TLR4-dependent, we treated macrophages from wild type and TLR4 knock out mice with 200 ng/ml of the different variants. LPS was used as positive control. As predicted, the IFN-β and IL-6-stimulating activity of iso2 in wild type macrophages (18±2 and 10±2.5 fold, respectively) was abolished in TLR4^-/-^ macrophages [13] (**Fig 3A and 3B**). Similarly, the newly isolated isoB and p7 variants activated IFN-β (18±3 and 278±30 fold, respectively) and IL-6 (11±2 and 119±20, respectively) mRNAs in wild-type, but not in TLR4^-/-^ macrophages indicating the reliance of TLR4-dependent pathways by recombinant ProTα variants for the induction of type I IFNs and IL-6 (**Fig 3A and 3B**). In contrast, the expression of TNF-α mRNA was induced in wild-type macrophages by all the variants (20±2, 17±3, and 45±5 fold for iso2, isoB and p7, respectively) and, although significantly reduced, remained readily measurable in TLR4^-/-^ macrophages (3±0.3, 6±0.1, and 10±2 fold for iso2, isoB and p7, respectively)(**Fig 3C**). In control LPS treated macrophages, TNF-α mRNA expression was increased 40±4.5 fold in wild-type macrophages, but completely abrogated in TLR4^-/-^ macrophages, confirming the sole utilization of TLR4 by LPS (**Fig 3C**). Taken together, these observations suggest that alternative receptor(s) and TLR4-independent pathways may be involved in the induction of TNF-α by ProTα variants in murine macrophages.

**Fig 4:**
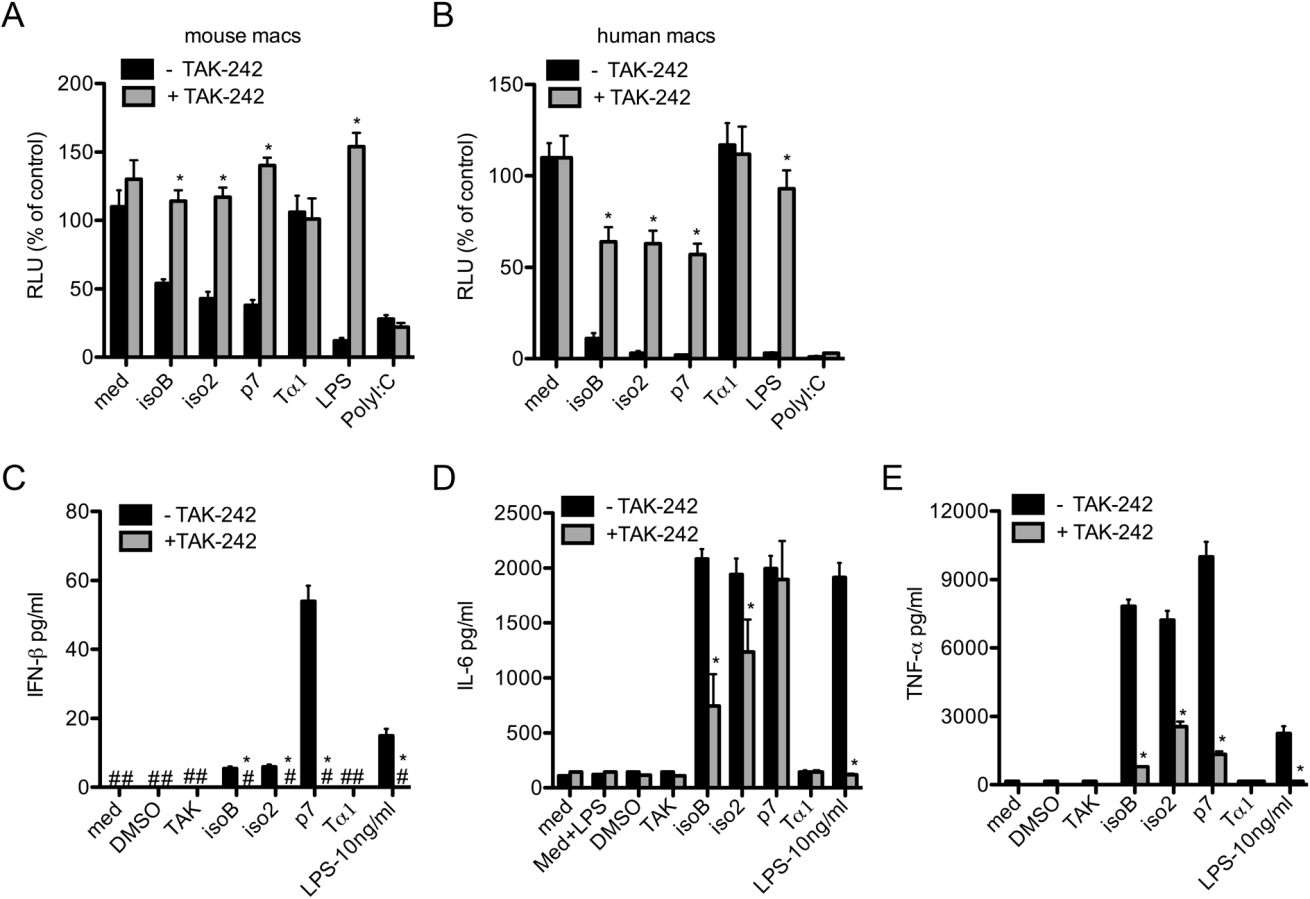
Anti-HIV-1 activity and cytokine release by ProTα variants are not entirely TLR4-dependent. (A) Murine or (B) human macrophages untreated or treated with the specific TLR4 inhibitor small molecule TAK-242 (3 μM) for 2 h, were stimulated with 20 ng of ProTα variants for 2 h and then infected with HIV-1_VSV_ (MOI: 0.01) expressing the Luciferase reporter gene_._ Anti-HIV-1 activity of ProTα variants was assessed on cell lysates 48 h post-infection by luciferase assay normalized for the protein concentration. Supernatants from infected human macrophages treated with ProTα variants in the presence or absence of TAK-242 were collected 6 hours and 48 hours post-infection and used to measure the release of (C) IFN-β, and (D) IL-6 and (E) TNF-α, respectively. Each experiment was repeated at least three times. Data represent mean ± SD. Student’s *t* test was used to compare means of treated versus control samples. *P<0.05 was considered to be statistically significant.

To determine whether IFN-β and TNF-α inducing activities of ProTα variants in human macrophages were similarly reliant on TLR4 signaling, we used a specific small molecule inhibitor of TLR4, TAK-242 [32]. We first evaluated the activity of the TLR4 inhibitor in normal mouse macrophages the presence of the TLR4 agonist LPS. Mouse macrophages pretreated with 3 μM of TAK-242 for 2 h were stimulated with the different ProTα variants at concentrations 20 ng/ml, or with control ultra-pure LPS (10 ng/ml and 100 ng/ml), or PolyI:C (5 μg/ml) for another 2 h and finally infected with VSV envelope-pseudotyped HIV-1 expressing the luciferase reporter gene, as we previously described [13]. The anti-viral activity was determined by luciferase assay on cell extracts 24 h post-infection. In the presence of the TLR4 inhibitor, the antiviral activity of LPS was abolished, while the anti-HIV-1 activity of the TLR3-dependent polyI:C remained intact, thus supporting the specificity of the inhibitor. Similarly to LPS, in mouse macrophages the anti-HIV-1 activity of all ProTα variants was reversed by the inhibitor, confirming their TLR4-mediated mechanism (**Fig 4A**).

Similar experiments were conducted in human macrophages to determine the TLR4 dependent activities of ProTα variants. As expected, the TLR4 inhibitor entirely blocked the antiviral activity of LPS in human macrophages 24 h post-infection with HIV-1_VSV_, while leaving that of PolyI:C unaffected. However, despite the complete abrogation of LPS antiviral response, TAK-242 lowered the ProTα variants’ activity only to 60%, further suggesting that in human macrophages a second receptor may be participating in the antiviral signals elicited by ProTα variants (**Fig 4B**).

Importantly, control recombinant Tα1 purified in parallel to the other ProTα variants using the same protocol did not show any activity on either murine or human macrophages (**Fig 4A and 4B**).

To determine the possible correlation between antiviral response and IFN-β, IL-6, and TNF-α, their secretion was measured in supernatants collected from human macrophage cultures 6 h and 48 h post-infection with HIV-1_VSV_. All three variants of ProTα stimulated the release of IFN-β by 6 h (isoB: 5.5±0.6 pg/ml; iso2:6±0.7 pg/ml; and p7:54±4.5 pg/ml) and IL-6 and TNFα by 48 h (isoB: 2083±89 pg/ml; iso2 1943±143 pg/ml; p7:1996±115 pg/ml, and isoB: 7841±292 pg/ml; iso2: 7215±41 pg /ml; p7 9998±659 pg/ml, respectively). Of ProTα variants, p7 displayed the most potent activity for all these cytokines (**Fig 4B–4E**). Blocking of TLR4 signaling pathway completely abrogated IFN-β production by all ProTα variants. However, while the LPS induction of TNF-α and IL-6 was completely blocked by the TLR4 antagonist, the secretion of TNF-α and, in particular, IL-6 by all three variants remained significant under the same TLR4 inhibitory conditions, strongly supporting the possibility that ProTα variants act also through TLR4 independent signaling (**Fig 4D and 4E**).

### ProTα variants are predicted to be intrinsically disordered proteins (IDPs) with different interactability

**Fig 5:**
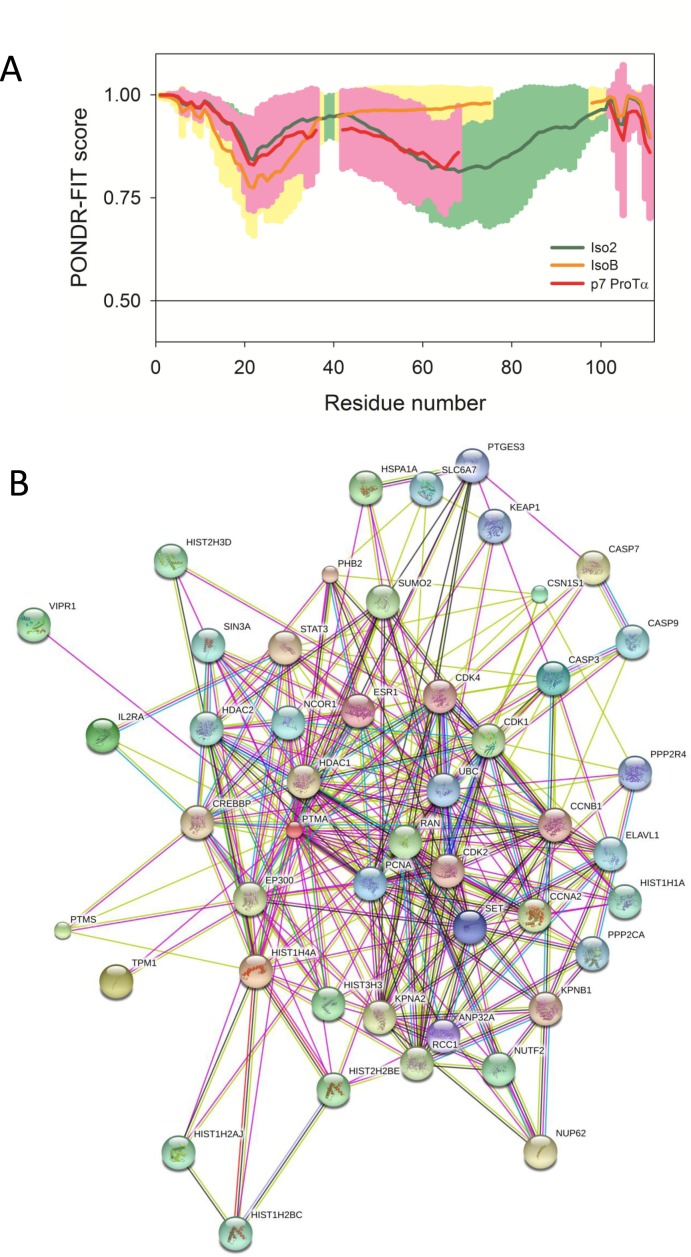
ProTα variants are predicted to be intrinsically disordered proteins with high binding promiscuity. (**A**) Per-residue disorder distribution in variants of human ProTα (green line and shade–iso2; yellow line and shade–isoB variant; red line and shade–p7 ProTα variant). Disorder propensity was evaluated by PONDR-FIT. Scores above 0.5 correspond to disordered residues/regions. Profiles were aligned to match some specific disorder-based feature. Shades represent distribution of errors in disorder estimation by PONDR-FIT. (**B**) Evaluation of the experimental and predicted interaction of the human iso2 (UniProt ID: P06454) by STRING (Search Tool for the Retrieval of Interacting Genes) [28], which produces the network of predicted associations for a particular group of proteins. The network nodes are proteins and the edges represent the predicted functional associations. An edge may be drawn with up to 7 differently colored lines each representing one of the seven types of evidence used in predicting the associations. A red line indicates the presence of fusion evidence; a green line—neighborhood evidence; a blue line–co-occurrence evidence; a purple line—experimental evidence; a yellow line–text mining evidence; a light blue line—database evidence; a black line–co-expression evidence [28].

To gain insight into the possible interactions of the ProTα variants with other proteins, we performed an in silico analysis of the proteins. Since amino acid sequences of IDPs possess several characteristic and recognizable features, these proteins are predictable from their sequences alone [33–36]. The per-residue intrinsic disorder propensity of human iso2 ProTα and its isoB and p7 variants was evaluated using the members of the PONDR family of intrinsic disorder predictors, PONDR^®^ VLXT, PONDR^®^ VL3, PONDR^®^ VSL2 and PONDR-FIT. In these analyses, scores above 0.5 correspond to regions/residues were predicted to be disordered. The overall disorder status of these proteins evaluated as averaged disorder scores of aforementioned disorder predictors are summarized in **Table 1**, and the PONDR-FIT-based per-residue disorder profiles of human iso2 and its variants are depicted in **Fig 5A**.

**Table 1:** Evaluation of functional disorder in human ProTα and its variants analyzed in this study.

Protein name	Disorder score^a^	Disorder-based binding sites	PTM sites^b^
iso2 ProTα	0.92±0.05	1–42; 45–58; 76–100	S2^A^; K20^S^; E25^C^; E26^C^; E28^C^; E40^C^; E42^C^; E43^C^; E46^C^; E48^C^; E52^C^; E55^C^; E56^C^; E57^C^; E58^C^; E59^C^; E62^C^; E63^C^; E64^C^; E65^C^; E66^C^; E67^C^; E68^C^; E69^C^; E73^C^; E74^C^; E75^C^; E79^C^; E81^C^; E82^C^; E84^C^; R90^R^; E93^C^; D98^PC^; D101^PC^; E109^C^
isoB ProTα	0.91±0.06	1–23; 36–86	S2^A^; K20^S^; E26^C^; R49^R^; S52^P^; R64^M^; R71^PC^; R77^PC^
p7 ProTα	0.92±0.05	1–52; 67–73	S2^A^; K20^S^; E25^C^; E26^C^; E28^C^; E37^C^; E38^C^; E41^C^; E43^C^; E47^C^; E50^C^; E51^C^; E53^C^; E54^C^; E57^C^; E58^C^; E59^C^; E60^C^; E63^C^; E64^C^; E66^C^; E68^C^; E69^C^; E70^C^

^a^Disorder scores were calculated as average scores of four disorder predictors, PONDR^®^ VLXT, PONDR^®^ VL3, PONDR^®^ VSL2, and PONDR-FIT

^b^Superscripts indicate different types of post-translational modfications: A–acetylation; C–carboxylation; M–methylation; P–phosphorylation; PC–proteolytic cleavage; R–ADP-ribosylation; S–SUMOylation.

The data shows that these proteins are highly disordered since their disorder plots are entirely located above the 0.5 threshold. Although experimental data on structural features of human isoB and p7 are not available yet, the highly disordered nature of human iso2 is well-established [6, 8]. Since disorder profiles of all proteins are rather similar, it is very likely that isoB and p7 ProTα variants are highly disordered as well.

Different post-translational modifications are also predicted for ProTα variants (**Table 1**). Disordered regions frequently contain sites of various post-translational modifications (PTMs), such as phosphorylation, acetylation, lipidation, ubiquitination, sumoylation, glycosylation, etc., that allow for the modulation of the disorder-based biological functions of IDPs [27, 37, 38]. Some proteins require multiple types of PTMs for their function. For such multi-PTM proteins, modified sites in proteins can not only mediate individual functions, but can also function together to fine-tune molecular interactions and to modulate overall protein activity and stability [27, 39, 40].

The data in this study suggest that human ProTα variants are multifunctional proteins that interact with different partners. To assess the potential interactions of human ProTα we used the STRING database, which is considered the most comprehensive of all information on functional links between proteins [28]. Version 9.0 of STRING (accessible at http://string-db.org) covers more than 1,100 completely sequenced organisms, including *Homo sapiens* (**Fig 5B**). This analysis showed that human ProTα is a highly promiscuous binder and that, as such, it should be considered as an intrinsically disordered hub protein. Unfortunately, data for isoB and p7 ProTα is not currently available at the STRING database. However, based on the overall high level of intrinsic disorder in these proteins and on the fact that all of them contain predicted disorder-based binding regions and multiple PTM sites, it is highly likely that all ProTα variants evolved to bind.

Furthermore, based on the analysis using the Eukaryotic Linear Motif resource [41], human ProTα protein is also predicted to have bipartite (residues 89–107) and monopartite (residues 102–108) variants of the classic positively charged nuclear localization sequences. These findings explain why this protein is found both inside and outside of the nucleus [42–44]. Although no nuclear localization signals were observed by ELM analysis in the isoB and p7 ProTα variants [41], it cannot be excluded that they may be translocated to the nucleus through interaction with other factors.

### ProTα variants are rapidly released in response to HIV infection

**Fig 6:**
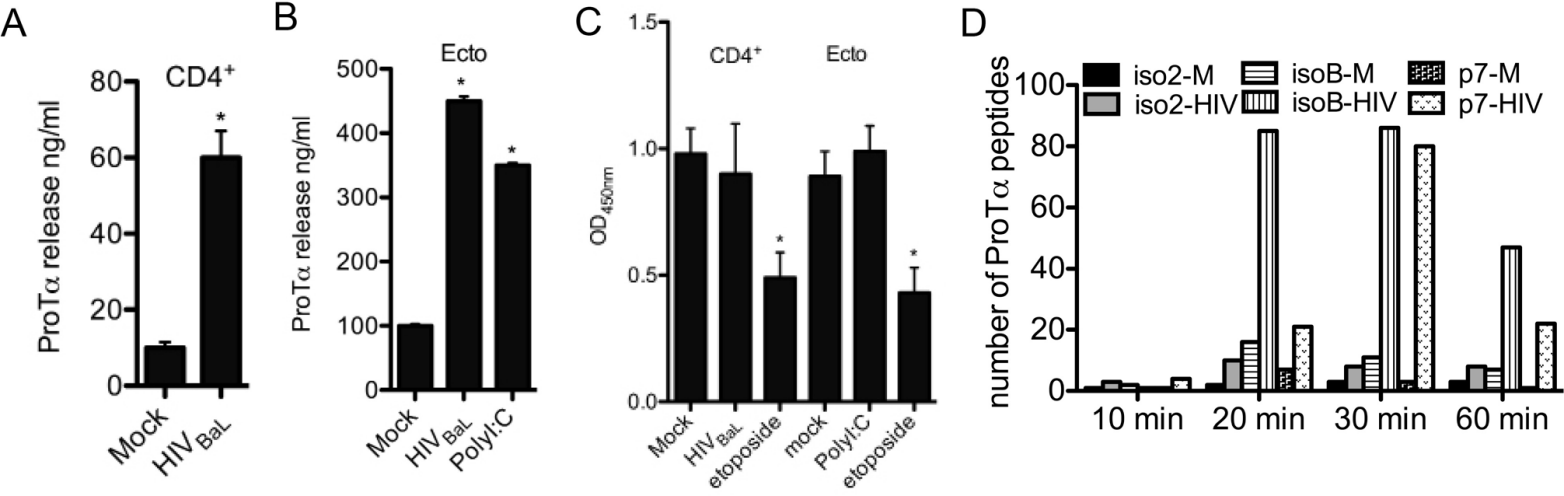
ProTα variants are rapidly released in response to HIV infection and PolyI:C treatment. Release of ProTα variants by ELISA (A and B) and control MTS cytotoxicity assay (C) were performed on 24 h supernatants from mock or HIV-1_BaL-_infected (MOI 0.01) primary human CD4^+^ T cells and ectocervical cells (Ecto). Cells treated for 24 h with the DNA-damaging agent etoposide (2 μM) were used as a positive control in the cytotoxicity assay (C). (D) Mass spectrometry analysis of the variants’ specific peptides present in the supernatants from healthy donor-derived PBMCs uninfected or 10, 20, 30, 60 min post-infection with HIV-1_BaL_ at MOI 0.01 showed the rapid release of the ProTα variants.

Several reports indicate that ProTα is rapidly released either in response to Pichinde virus infection or in response to neuronal damage [9, 45]. Therefore, we asked whether HIV-1 infection similarly affected the release of ProTα variants. Infection of primary human CD4^+^ T cells with HIV-1_BaL_ triggered the release of ProTα at 24 h post-infection (60 ng/ml in HIV-1-infected *vs*. 10 ng/ml in mock-infected supernatants) (**Fig 6A**). As we first observed the presence of ProTα variants in the cervicovaginal lavage fluids, we asked whether the release of ProTα could be triggered in ectocervical cells 24 h after the infection with HIV-1. Significant release of ProTα could be readily measured in the supernatants of HIV-1 infected ectocervical cells (from 100 ng/ml to 450 ng/ml in the presence of the virus) (**Fig 6B**). Parallel treatment of ectocervical cells with the TLR 3 ligand PolyI:C similarly induced the release of ProTα (from 100 ng/ml to 350 ng/ml). No difference in mock and HIV-1 infected cells viability 24 h post-infection was observed, confirming that the release of ProTα was not due to cell death (**Fig 6C**).

Despite showing the infection-dependent release of ProTα, these studies were limited by the inability of the ELISA assay to distinguish the different variants. We therefore determined the kinetic of release upon HIV-1_BaL_ infection and whether all variants were equally released. Healthy donor PBMCs were infected with HIV-1_BaL_ and supernatants were collected at 10, 20, 30, 60 min post-infection. The accumulation of p24 (500 ng/ml) in control HIV-1_BaL_-infected PBMC after 7-day culture confirmed the proper viability and replication of the virus (data not shown). Supernatants were subjected to mass spectrometry analyses in a positive and negative mode to identify the amino acid sequences and the number of the variants’ specific peptides released in response to HIV-1 infection (**Table 2**). We could detect an increase in the number of isoB specific peptides (from 16 in medium to 85 in HIV-1 infected supernatant) and p7 specific peptides (from 7 in medium to 21 in HIV-1 infected supernatant) as soon as 20 minutes post-infection (**Fig 6D**). The number of both ProTα variants isoB and p7 peptides in HIV-1 infected supernatants increased with time within the first 30 minutes (isoB: 86 and p7: 80), but dropped thereafter (isoB: 47 and p7: 22) (**Fig 6D**). Interestingly, the number of released Iso2 specific ProTα peptides in response to HIV-1_BaL_ was much lower then isoB and p7 (from 2 in medium to 10 in HIV-1 infected supernatant at 20 minutes post-infection), thus suggesting that only ProTα variants without NLS are rapidly and preferentially released in response to HIV-1 infection (**Fig 6D)**.

**Table 2:** Mass spectrometry identification of the amino acid sequences and frequency of peptides (in brackets) released by PBMCs in response to HIV infection.

ProTα	Infection	10 min	20 min	30 min	60 min
Iso2	Mock	tdedd (1)	satgkraa (1); dgeeedgdedee (1)	satgkraa (2); dapangnaen (1)	satgkraa (3)
Iso2	HIV-1	aaeddedddvdt(2); tdedd(1)	aaeddedddvdt (3); satgkraa (1); dapangnaen (2); dtkkqktdedd (3); dgeeedgdedee (1)	satgkraa (2); dapangnaen (1); dtkkqktdedd (3); dgeeedgdedee (2)	satgkraa (3); dapangnaen (2); dtkkqktdedd (2); dgeeedgdedee (1)
IsoB	Mock	lsqlr (2)	mtmsipr (3); lsqlr (5); asgqlk (5); memk (1); rptr (2)	mtmsipr (5); lsqlr (1); asgqlk (2); memk (3)	mtmsipr (4); lsqlr (2); asgqlk (1)
IsoB	HIV-1	mtmsipr (1)	dapangnavr (10); mtmsipr (15); lsqlr (17); asgqlk (5); memk (1); rptr (2); mtmsiprlsqlr (12); tmsiprsrrpt (3);qlrasgq (20)	dapangnavr (15); mtmsipr (17); lsqlr (20); asgqlk (5); memk (12); mtmsiprlsqlr (10); tmsiprsrrpt (13); qlrasgq (10)	dapangnavr (10); mtmsipr (7); lsqlr (12); asgqlk (5); memk (1); mtmsiprlsqlr (5); msiprsrrpt (7)
P7	Mock	geqeadnev (1)	geqeadnev (1)	gegeeegwr (2)	gegeeegwr (1)
P7	HIV-1	dapadeeng (3)	gegeeegwr (6); dapadeeng (8)	gegeeegwr (27); dapadeeng (22)	gegeeegwr (10); dapadeeng (5)

## Discussion

Here we show that similar to iso2 ProTα [13], the recently isolated new isoB and p7 ProTα variants potently induce type I and type III IFNs in primary human macrophages through the activation of TLR4. The immunomodulating activities of ProTα variants extended to the stimulation of IL-6, TNF-α and RANTES and in all instances, the new variants p7 and isoB appeared to be relatively more potent than Iso2 (p7>isoB>iso2). We also showed that the antiviral response and the stimulation of IFN-β and IL-6 gene expression by all ProTα variants is completely abrogated in murine TLR4^-/-^ macrophages, whereas the induction of TNF-α is only partially reversed. Similarly, experiments with TAK-242, a specific small molecule inhibitor of TLR4, showed that in conditions of complete suppression of LPS responses, the antiviral activity elicited by the ProTα variants was significantly, but not entirely, reversed in human macrophages. TLR4 inhibition in human macrophages showed variable effects on the secretion of the tested cytokines. While IFN-β secretion was completely blocked in the presence of the inhibitor, TNF-α release was only partially, though significantly, reduced. On the other hand, TAK-242 only partially blocked IL-6 release in iso2 and isoB treated human macrophages, but did not affect p7 treated cells. Overall these observations indicate that, similarly to iso2, both isoB and p7 can signal through TLR4. However, these interactions may not be exclusive and the differential response to the variants suggests that they possess independent specific activities. The incomplete antiviral response and suppression of IL-6 and TNF-α in human macrophages could be accounted for by the differential effect of the inhibitor on different cell types, although, based on the predicted structural characteristics of these proteins, the differential response more likely results from the interaction of ProTα variants with signaling molecules other than TLR4. The nature of these signaling molecules remains unclear and may be completely independent of the pathways immediately downstream of TLR4.

The in silico analysis predicted that the disordered structure of ProTα variants may indeed favor their interaction with multiple binding partners. The correlations found between predicted disorder and functional sites of proteins are not too surprising since recent studies indicated that intrinsic disorder is vitally needed for the functionality of many proteins, and that IDPs and hybrid proteins with functional disordered regions are very common in any given proteome [45–49]. The high intrinsic disorder levels, the presence of several disorder-based binding regions and the existence of numerous PTM sites suggest that human iso2 and its isoB and p7 variants are promiscuous binders whose interactions may be controlled by post-translational modifications [27, 37, 38]. Although our bacteria-produced recombinant proteins lack such modifications, post-translational changes may be required for the fine regulation of the native forms. Comparative analysis of the biological properties of native *vs*. recombinant ProTα will be required to confirm this possibility and further work will be necessary to identify the alternative interactors of ProTα variants.

It has become increasingly evident that damage associated molecular patterns (DAMP) play a role in the control not only of cancer, but also of viral replication [46, 47]. DAMPs are molecules produced from injured or activated cells and can induce an inflammatory response through TLR activation [48]. Our findings that iso2, isoB and p7 are released within minutes from viral infection along with data from other laboratories showing that ProTα is secreted upon cellular stress, strongly suggest that these short proteins behave as DAMPs that may function to protect against ischemia, cancer or viral infection [9, 15, 45, 48]. The role of the isoB and p7 variants as DAMPs is further supported by the established iso2 direct TLR4 binding and activation of the TLR4-TRIF pathway [13, 16]. As such, the release of ProTα variants may be a signature of the anticancer and/or antiviral innate immune response.

Taken together our data indicate that ProTα variants function as immunomodulating agents. TLR ligands can be important tools to orchestrate not only anti-viral, but also anti-tumor responses by stimulating anti-tumor immunity [49]. Besides being expressed in immune cells, functional TLRs are present on many cancer cells, but their contribution to anti-tumor response has been controversial [50, 51]. Immunomodulatory stimulation of cancer microenvironment could be particularly important to overcome the release of soluble factors that inhibit the generation and maturation of DCs and while increasing the accumulation of myeloid suppressor cells [52, 53]. Furthermore, the intratumoral expression of chemokines may be a mechanism to recruit immune cells with anti-tumor activity. For instance, the induction of RANTES by ProTα variants may play a role in the selective recruitment of memory T-cells [54]. It will be important to evaluate whether the TLR4-mediated IFN-β induction by the isoB and p7 variants may be an efficient immunotherapeutic approach to promote DC maturation and activation.
